# Lifestyle change in Kerala, India: needs assessment and planning for a community-based diabetes prevention trial

**DOI:** 10.1186/1471-2458-13-95

**Published:** 2013-02-01

**Authors:** Meena Daivadanam, Pilvikki Absetz, Thirunavukkarasu Sathish, K R Thankappan, Edwin B Fisher, Neena Elezebeth Philip, Elezebeth Mathews, Brian Oldenburg

**Affiliations:** 1 Achutha Menon Centre for Health Science Studies, Sree Chitra Tirunal Institute for Medical Sciences and Technology, Trivandrum, Kerala, India; 2 Division of Global Health (IHCAR), Department of Public Health Sciences, Karolinska Institutet, Nobels vag 9, Stockholm, 171 77, Sweden; 3 National Institute for Health and Welfare, Helsinki, Finland; 4 School of Public Health and Preventive Medicine, Monash University, Melbourne, Australia; 5 Department of Health Behaviour and Health Education, Gillings School of Global Public Health, University of North Carolina at Chapel Hill, Chapel Hill, USA

**Keywords:** Diabetes mellitus, Real world intervention, Diabetes prevention, Pre-diabetes

## Abstract

**Background:**

Type 2 Diabetes Mellitus (T2DM) has become a major public health challenge in India. Factors relevant to the development and implementation of diabetes prevention programmes in resource-constrained countries, such as India, have been under-studied. The purpose of this study is to describe the findings from research aimed at informing the development and evaluation of a Diabetes Prevention Programme in Kerala, India (K-DPP).

**Methods:**

Data were collected from three main sources: (1) a systematic review of key research literature; (2) a review of relevant policy documents; and (3) focus groups conducted among individuals with a high risk of progressing to diabetes. The key findings were then triangulated and synthesised.

**Results:**

Prevalence of risk factors for diabetes is very high and increasing in Kerala. This situation is largely attributable to rapid changes in the lifestyle of people living in this state of India. The findings from the systematic review and focus groups identified many environmental and personal determinants of these unhealthy lifestyle changes, including: less than ideal accessibility to and availability of health services; cultural values and norms; optimistic bias and other misconceptions related to risk; and low expectations regarding one’s ability to make lifestyle changes in order to influence health and disease outcomes. On the other hand, there are existing intervention trials conducted in India which suggests that risk reduction is possible. These programmes utilize multi-level strategies including mass media, as well as strategies to enhance community and individual empowerment. India’s national programme for the prevention and control of major non-communicable diseases (NCD) also provide a supportive environment for further community-based efforts to prevent diabetes.

**Conclusion:**

These findings provide strong support for undertaking more research into the conduct of community-based diabetes prevention in the rural areas of Kerala. We aim to develop, implement and evaluate a group-based peer support programme that will address cultural and family determinants of lifestyle risks, including family decision-making regarding adoption of healthy dietary and physical activity patterns. Furthermore, we believe that this approach will be feasible, acceptable and effective in these communities; with the potential for scale-up in other parts of India.

## Background

### Type 2 Diabetes Mellitus is a major public health issue in India

Next only to China, India has the second largest number (>61 million) of individuals with Type 2 Diabetes Mellitus (T2DM) in the world and this is expected to nearly double by 2030 [1]. The national T2DM prevalence in 2011 was already 8.3 percent [1]. Furthermore, a large proportion of individuals are at “high risk” of progression to diabetes [1] which occurs more quickly than in most developed countries [2]. These observations, together with the high rates of complications and mortality [3] associated with T2DM, demonstrate that diabetes prevention should be an urgent priority for the government and other organizations in India.

### Evidence supports lifestyle interventions for diabetes prevention

A number of efficacy trials from China [4], Finland [5], U.S.A. [6], India [7] and Japan [8] provide strong empirical support for lifestyle change programmes in preventing T2DM among individuals with impaired glucose tolerance (IGT). Reduction in T2DM incidence ranged between 42-58% in these various trials [5,7,9-12], with generally good maintenance for up to 20 years [4]. Moreover, behavioural interventions have been shown to be more cost-effective than drug treatment [13,14], particularly when delivered via group-based intervention programmes [15]. A series of ensuing implementation trials conducted in different countries in recent years [7,16-18] have now also demonstrated that the findings from efficacy trials can be replicated in ‘real world’ community settings with more feasible, acceptable and cost-effective delivery systems [19,20] and with similar outcomes [5,16,21-24].

### Transferability and uptake in resource poor settings requires critical evaluation

The majority of these efficacy and implementation trials – except the Da-Qing study in China and the Indian Diabetes Prevention Programme (IDPP) – were developed and delivered in developed countries that are very different from rapidly developing countries in terms of their health systems, culture, traditions and lifestyle behaviours related to nutrition and diet. Most of these programmes were also undertaken in countries, where there was quite a strong enabling environment of policy and other supports for the prevention and control of chronic non-communicable diseases (NCDs) aimed at increasing population awareness of lifestyle-related risk [25,26]. These programmes have typically focused on weight loss, greater intake of fiber, reduced total and saturated fat and increase in daily physical activity [5]. Most of these programmes have also used behaviour change approaches and techniques that have emphasised outcome expectations, self-efficacy, setting of individual goals and creation of specific action plans in order to achieve lifestyle change in key behaviours. However, the socio-behavioural approaches and models on which these strategies are based have also been largely derived from health behaviour theories and models that have been primarily developed in Europe and the United States [27,28]. Currently, there is little research concerning the factors that influence the feasibility and adaptation of T2DM prevention programmes to more resource constrained countries, cultures and settings [29].

The state of Kerala in India has high literacy (90.9%) and is the most advanced in terms of demographic and epidemiological transition, with the largest proportion of elderly and those suffering from NCDs, including diabetes [30,31]. However, as is the case in most of India, the majority (76%) of Kerala’s 33 million inhabitants reside in non-urban areas [31]. These factors make rural Kerala an ideal setting in which to trial and develop new approaches to T2DM prevention as commentators have suggested that the rest of India is likely to become like Kerala in the future [30]. In other words, interventions developed and evaluated in Kerala have the potential to be adopted elsewhere in India in the future.

This paper describes the findings from a needs assessment for a diabetes prevention programme in Kerala by triangulating and synthesizing the evidence from three different sources:

1) A review of empirical studies relevant to understanding lifestyle risk factors, their determinants and lifestyle interventions for diabetes prevention in India and more specifically, Kerala.

2) A review of policy and other documents relevant to diabetes prevention in India and Kerala.

3) A focus group study concerning lay perceptions of T2DM and its prevention in Kerala.

Our aim is to show how these findings would inform the design and delivery of the intervention programme for the Kerala Diabetes Prevention Programme (K-DPP).

## Methods

The methodology and conduct of data collection and analysis for each of the three data sources are described below. The needs assessment study was carried out between February 2010 and April 2012, when the final update for the literature searches was conducted.

### Review of empirical studies

Research literature search was confined to studies related to diet, physical activity and tobacco use in relation to non-communicable diseases, their risk factors and determinants in India and Kerala in particular, identified through the PubMed database. Four searches were conducted in the Pubmed database using MeSH terms related to diet, physical activity, tobacco use and health promotion interventions in India. Tobacco use was included as it has recently been recognised as a risk factor of T2DM [32]. Complete search strategy and search results are presented in Additional file 1. All available abstracts were reviewed and relevant articles were obtained through the Internet or directly from the authors. Publications that were unavailable are not included in the review. Our focus was on identifying relevant epidemiological studies concerning the major social and behavioural risk factors and non-pharmacological intervention studies. For the intervention studies, we applied the following inclusion criteria: dietary, physical activity, tobacco-related or lifestyle modification interventions conducted in India among adult population (>/= 19 years) looking at individual risk factor modification alone or in combination with life-style modification for diabetes, cardiovascular diseases or NCDs and published in the last 10 years. Nine intervention studies were identified and two could not be accessed even after writing to the authors. The remaining seven studies are listed in Table 1.

**Table 1:** Details of completed non-communicable disease intervention studies in the Indian context

**Study (Author)**	**Target population**	**Study design**	**Intervention**		**Study outcomes**
**Target risk factor**			**Components**	**Response**	
**1. Indian Diabetes Prevention Programme (IDPP) 1&2 (Ramachandran A et al. 2010)**: Risk for and incidence of T2DM	- Follow-up of 845 out of 869 IGT subjects from IDPP 1and 2 studies,recruited from clinic setting followed up for 3 years	- 3 yr RCT	*Individual:*	- IDPP 1: 502 out of 531 (94.5%) participants found to have IGT after standard Oral Glucose Tolerance Test (OGTT)	- IDPP-1: Decrease in relative risk 29% (LSM), 26% (Metformin) & 28% (LSM+Metformin)
		- IDPP 1: 4 groups	- Personal sessions at 6-month intervals		
		1) Control with standard advice:	- 0.15-0.75 h/year by dietician & social worker & monthly telephone contacts		
		2) LSM	- LSM: diet & physical activity modification		
		3) Metformin (500 mg/day)			- IDPP-2: Cumulative incidences at 36 months: 30% (LSM +Pioglitazone) & 32% (LSM+placebo)
	- 2 groups of participants: Group 1 (n=667): Baseline isolated IGT; Group 2 (n=178): IGT+IFG	4) LSM + Metformin			
		- IDPP 2: 2 groups		- IDPP 2: 367 out of 407 (90.2%) participants found to have IGT after standard OGTT	
		1) LSM + placebo			- No additional benefit with drugs
		2) LSM + Pioglitazone (30 mg/day)			
**2. Diabetes Prevention & Management (DPM) programme (Balagopal P et al. 2008)**: Proportion with high fasting blood glucose levels	850 village inhabitants, comprising adults and youth aged 10–92 years (included healthy, impaired fasting glucose and T2DM individuals)	7-month community-based non-pharmacological lifestyle intervention	*Individual:*	- Total eligible residents: 950	FBG levels decreased by 3% (healthy adults), 11% (adults with IFG), 17% (youth with IFG) & 25% (adults with T2DM)
			- 10 face-to-face interviews		
				- Baseline survey: 850	
			*Group:*		
				- Post-intervention survey: 703 (Attrition rate due to migrations & refusals: 17%)	
			- Culturally sensitive sessions on physical activity & diet		
			*Community:*	- Response rate at baseline: 89.5%	
			- Participatory analysis of village		
			- Involvement of village leaders, peer educators & residents		
**3. Chennai Urban Population Study-17 (Mohan V et al., 2006):** Physical inactivity	All individuals above age of 20 living in two residential colonies of urban Chennai	Community-based intervention for increasing physical activity. Baseline cross-sectional survey and a 7-yr follow-up cross-sectional survey.	*Individual:*	- Baseline cross-sectional survey (1996): 479 out of 524 eligible participants (91.4%) - 7-yr follow-up cross-sectional survey (2004): 705 out of 712 eligible participants (99%)	- Proportion of light-grade activity reduced in both men (55% to 36%) and women (74% to 57%) - Proportion of residents exercising increased from 14% to 59% - Community’s response: residents mobilised resources and constructed a park.
			- Culturally tailored education campaign & materials, social worker visits - Diabetes and high risk intervention: information on diabetic status & individual counseling *Population:* - Awareness programme using public lectures, video clippings & short skits		
**4. Community-based intervention in Ballabgarh, India (Krishnan A et al. 2010):** Non-communicable disease risk factors	Residents in urban areas of Ballabgarh block, Faridabad district, Haryana (near New Delhi)	- Community-based demonstration project using the Health Settings approach. - Cross-sectional surveys at pre-intervention and 3-year follow-up: pre-intervention survey in 2003-04 and post-intervention survey in 2006-07	*Individual:* - Advocacy and medication - Individual empowerment *Community:* - Social enhancement and community empowerment - Reorientation of health services	Not mentioned	- Programme reach (proportion of community who came in contact with the programme): 25% - Change from baseline proportion: consuming < 5 servings of fruits and vegetables decreased by 3% (men), 5% (women); Elevated BP decreased 9% (men), 2% (women)
**5. Work site intervention programme on cardiovascular risk factors (Prabhakaran et al. 2009):** Cardiovascular risk factors	Employees and their family members (age 10–69 years) from 10 centres (Bangalore, Coimbatore, Delhi, Dibrugarh, Hyderabad, Lucknow, Ludhiana, Nagpur, Pune and Trivandrum)	Work site demonstration project: - Intervention sites: Baseline cross-sectional survey, 4-year health intervention programme and a repeat cross-sectional survey. - Control sites: Baseline cross-sectional survey, 4-year minimal interventions and a repeat cross-sectional survey.	*Individual:* - One-on-one interactions between the trained health project personnel and the participants *Group:* - Dynamic group interactions and healthy meals *Population or community:* - Use of posters, banners at strategic locations in the industry	- Baseline cross-sectional survey: Intervention sites: 82.4% and control site: 90.0% - Repeat cross-sectional survey: Intervention sites: 98.3% and control site: 90.7%	Change in proportion of risk factors in intervention vs. control sites: tobacco use: 39% to 29% vs. 17% to 20%, extra salt use: 28% to 13% vs. 22% to 25%, median physical activity score: 6 to 11 vs. 8 to 6, fruit consumption: 38% to 45% vs. 36% to 38%



			- Handouts, booklets and video films shown on the internal cable network		
**6. Community-based intervention for tobacco cessation in rural Tamil Nadu, India: A cluster randomised trial (Kumar MS et al. 2012):** Tobacco use (smoking and smokeless tobacco)	Men aged 20–40 years using any form of tobacco who were residing in Tiruchirapalli district, Tamilnadu.	A cluster randomised trial with two months follow up.	*Group:*	- Attendance in first intervention session: 88.5%; second intervention session: 60.5%. The follow-up rates for intervention and control arms were 90.5% and 92.5%, respectively.	At 2 months:
			Two sessions of health education was offered by a health professional, five weeks apart, along with self-help material on tobacco cessation to intervention group. The control group received only self-help material.		- Self-reported point prevalence abstinence: 13% (intervention), 6% (control)
					- Quit attempt: 27% (intervention), 20% (control)
					- Harm reduction: 22% (intervention) 9% (control)
**7. Government of India smokeless tobacco campaign (Murukutla N et al. 2011):** Tobacco use	Individuals aged 16–50 years in urban and rural areas who are current smokeless tobacco users and have access to mass media (television or radio)	The six weeks campaign (November and December 2009) was evaluated with a nationally representative household survey of 2898 smokeless tobacco users during 20 December 2009 to 10 January 2010.	*Population:*	Screening interviews were completed in 92% of the respondents	- Awareness of the campaign: 63% (smokeless-only users), 72% (dual users)
			- An oral cancer surgeon from a tertiary care hospital in Mumbai described the serious illnesses and disfigurement of his patients, caused by cancers resulting from use of smokeless tobacco.		
					- Concern about their habit: 75% (smokeless-only users), 77% (dual users)

Abbreviations: *T2DM* Type 2 Diabetes Mellitus; *IGT* Impaired Glucose Tolerance; *IFG* Impaired Fasting Glucose; *RCT* Randomised Controlled Trial; *LSM* Life Style Modification; *FBG* Fasting Blood Glucose; *BP* Blood Pressure.

### Review of policy and other relevant documents

Policy documents related to national and state capacity, legislation, programmes and guidelines for NCD prevention and control, diet, physical activity, tobacco and alcohol were searched using Google search engine from the official websites of related government departments in India (Ministry of Health and Family Welfare, Government of India; Government of Kerala; and National Rural Health Mission). Initially the documents were accessed and reviewed by two of the authors (MD, PA). The list was then counter-checked for completion by members of the research team in Kerala (KRT, ST, NEP, EM). The reality of the reports in terms of actual work going on in Kerala was corroborated through the State Nodal Officer (NCD) under National Rural Health Mission (NRHM) in Kerala and the Additional Director of Health Services (Public Health), Directorate of Health Services, Government of Kerala.

### Conduct of focus groups

A sub-group of individuals with pre-diabetes (fasting blood glucose 110–125 mg/dl), who had participated in an earlier community-based survey in Trivandrum District in 2007–2008 and residing in rural areas [33], were invited by telephone to participate in a focus group discussion (FGD) related to their views on T2DM and its prevention. Of 84 individuals, 37 had incorrect phone numbers, 13 did not respond and 16 refused to participate, resulting in 18 participants in three FGDs. The first two FGDs were conducted separately among men (n=6; age: median: 54 yrs; range: 35–64 yrs) and women (n=6; age: median: 48 yrs; range: 33–63 yrs) with focus on the community’s understanding of diabetes, sources and use of health information, and interest in and feasibility and acceptability of interventions. The third FGD (n=6; age: median: 59 yrs; range: 40–64 yrs.) had an equal number of male and female participants and built on the findings from the previous FGDs to understand more about issues related to programme implementation and delivery.

An interview guide (Additional file 2) was prepared to help the moderator (MD) cover four main areas during the focus group discussion: 1) understanding of diabetes and level of interest to know more; 2) health information sources and access to them; 3) motivation to participate in a community-based diabetes prevention programme; and 4) how such a programme should be delivered and by whom? The FGDs were conducted in a health center run by a local non-governmental organization (NGO), *Health Action by Peopl*e (HAP) who conducted the original research [33]. Ethical clearance was obtained from the institutional review boards of Monash University, Melbourne, Australia and Sree Chitra Tirunal Institute for Medical Sciences and Technology, Trivandrum, Kerala for the K-DPP study and its development. Moreover, telephone invitations to participate in the FGDS were issued only after individuals with pre-diabetes were identified and approached by members of the NGO and informed about the present study. In addition, all participants gave informed consent before the FGDs were conducted. The FGDs were recorded on tape and transcribed verbatim before translation into English. Manifest content analysis [34] was performed using a combination of manual and software methods (Weft QDA, version 1.0.1.). Meaning units were initially identified from the text, condensed and coded; then interlinked to identify the main themes.

### Triangulation and synthesis of findings from different sources

Triangulation was used to integrate multiple data sources to improve the understanding of the diabetes problem in Kerala; to strengthen our interpretations; and to guide our decision-making to address the problem with an intervention based on available evidence [35]. In our synthesis, research literature corresponds to the currently available evidence base; policy documents reflect the policies and the programmes that underpin the public health practice related to NCDs in India; and the FGDs help inform our understanding of what people from the community really think about diabetes prevention, and how effective and realistic intervention strategies can be developed and tested.

## Results

### Review of empirical studies

#### Risk factors

Indian states show a wide variation in NCD prevalence, with Kerala ranking at the top. In comparison to the 8% age-adjusted prevalence of T2DM (fasting blood glucose >126 mg/dl or on medication) for all of India, the prevalence in Kerala ranged by gender and area from 12.3% among urban men to 22.2% among rural women. The state average is 14.3% for men and 17.8% for women. Respective figures are 51.4% and 61.5% for hypercholesterolemia (total cholesterol ≥200 mg/dl); 33.9% and 31.6% for hypertension (JNC VII); and 23.9% and 37.5% for overweight (BMI ≥25.0 kg/m^2^) [30].

#### Health behaviours

Smoking prevalence in Kerala is 28% among adult men (24% all India) [36], but almost non-existent among women. Unhealthy dietary habits are difficult to measure and compare due to the differences in definitions and the wide regional variation in composition of foods and diet found in India. Import–export data and national consumption and expenditure surveys suggest that more traditional (healthier) dietary patterns are being replaced by energy-dense (unhealthier) foods and beverages [37]. Between 1973 and 2005, energy derived from fats in India increased by 6% while energy derived from carbohydrates decreased by 7%. However the overall decline in energy from carbohydrates masked important and more specific changes; intake of coarse cereals, pulses, fruits and vegetables is inadequate and decreasing while consumption of refined carbohydrates, sugars, oils, fats, meat products and salt is increasing [37].

Physical inactivity and sedentariness among both genders is very common in India. Moreover, physical activity is related to occupation with hardly any reported spare-time activity [38]. In rural India, over 90% of the population report no vigorous or moderate physical activity during leisure-time [39]. Surprisingly, over 80% report no moderate or vigorous activity during work as well. The explanation for this is that even among those engaged in manual labour, physical activity does not really reach the required levels as work availability is erratic and seasonal and physical activity during non-work or leisure time is almost nil. Older or urban individuals, and women report more sedentary behaviour [40]. Apart from sleeping, TV viewing is the main spare-time activity [40,41]. Figures for different types of physical activity in Kerala are from early 2000, when the proportion of adults (30–70 yrs) physically inactive was 31% during work, 74% during leisure-time, and 39% during travel [42]. However, a study conducted in 2012 suggests considerably higher figures for leisure-time inactivity (98%) [Mathews E, unpublished data]. Indirect evidence from national consumption surveys (2009–2010) also reflects this high sedentariness, as Keralites spend five times more money on TVs, 10–15 times more on motorcycles and 430–1250 times more on cars as compared to the rest of India [43].

Determinants of health behaviours have been little studied in India. A quantitative study on an urban population in Southern India reported low levels of knowledge regarding the benefits of healthy lifestyle, causes of diabetes, and measures to prevent or manage the disease, especially among women*,* labourers and unskilled workers, and those with lower education [44]. Management of diabetes is inadequate, not only due to poor medical control [45], but also to factors such as people’s reluctance to share information of their illnesses even with significant others [46].

A study conducted in 2012 in Kerala identified several factors positively associated with physical activity, including role models and support among family, friends and neighbours; knowledge and advice from professionals; and presence of risk factors or chronic conditions in self or family. Environmental factors - poor access to facilities and heavy traffic - had negative associations [Mathews E, Pratt M & Thankappan KR, unpublished data].

Only a few studies have reported on determinants of dietary behaviours among Indians. Negligible associations were found between diet and social cognitions such as self-efficacy and outcome expectations [47,48]. For example, Sharma and colleagues observed a small but significant positive association between self-efficacy and eating of fruit and vegetables. Suggested explanations for the low associations were high dependency on family in health behaviour decisions [47] and a tendency to fatalism [48].

Qualitative findings from a recent large interview study from Kerala reveal many ways in which the culture enforces unhealthy lifestyle; influencing dietary practices, physical activity, as well as the taking of medications. “A protruding belly speaks of a life of embodied satisfaction – good social relationships, status, success and health” [46], p. 270. Even amidst worry about health and recognition of the risks of unhealthy eating, dietary adjustments are not made because refusing food would be seen as an expression of anger or annoyance, or as a sign of illness. As a compromise, “taking medicines (…) is palatable because it doesn’t disrupt the flow of food, care, love and pleasure in the households” [46], p. 270. Furthermore, leisure-time physical activity is seen as harmful in depleting one from energy needed for work, and it is especially discouraged among females who should focus on household chores and cooking instead [46]. This expectation is so strong that it prevails even in the younger generation after immigration into a culture that emphasizes fitness [49].

#### Community-based interventions

While a lot of recent medical and epidemiologic research in India has focused on NCDs [50], only a small proportion of the published studies have involved non-pharmacological interventions and only a few of these have included controlled study designs [51]. The seven interventions described in Table 1 targeted both normal population and high risk or diseased individuals, in clinical settings and ‘real world’ community settings. Only two of the studies [52,53] used a randomised controlled trial design, many lacked a control group [22,54-56] and none reported an explicit theory-base for behaviour change despite evidence supporting the use of theory [57]. Characteristic of many studies is inclusion of multiple components operating on different levels – individual, group, community and population – a factor likely to have influenced the outcomes positively [58]. The Diabetes Prevention and Management programme is one prime example combining community and individual empowerment. It was participatory, and included village leaders, peer educators and residents. Despite a short 7-month duration, the intervention outcomes were impressive in terms of fasting blood glucose levels across healthy, high risk and T2DM individuals. The work site intervention programme on CVD risk factors [59] also operated on multiple levels and achieved positive outcomes on multiple risk factors and health behaviours. Overall, the changes found in health behaviours and risk factors were small to moderate, but tended to be greater among those with higher risk. Furthermore, most studies had short follow-ups, insufficient to determine long-term maintenance of the outcomes.

The public health effect of these programmes is determined as much by their reach to the target population as by their effectiveness. Most studies (Table 1) show high response rates among those invited to the study, but total reach is rarely reported. As an exception, Krishnan et al. [55] reported a 25% reach for their community programme in spite of a multi-pronged approach and mass awareness campaigns.

### Policy document review

India’s history of national health policy and strategies addressing NCDs is a recent one. It started with the principal comprehensive law governing tobacco control in India, the Cigarettes and Other Tobacco Products (Prohibition of Advertisement and Regulation of Trade and Commerce, Production, Supply and Distribution) Act (COTPA), 2003 [60]. However, poor implementation and monitoring prevented the public from enjoying full benefits of the law [61]. In 2004, India became a party to the WHO-Framework Convention on Tobacco Control (FCTC), subsequently leading to *the National Tobacco Control Programme* (NTCP, 2007–12) [62], a Government initiative to facilitate the implementation of anti-tobacco laws, bring about greater awareness on the harmful effects of tobacco, sensitize all the stakeholders and fulfill the obligations under the WHO-FCTC. Kerala joined the programme in the first wave of the roll out.

From the perspective of T2DM prevention, two subsequent Government initiated programmes are particularly significant: the National Rural Health Mission (NRHM) [63] launched in 2005, and the National Programme for Prevention and Control of Cancer, Diabetes, Cardiovascular Diseases and Stroke (NPCDCS) [64] in 2008. The NRHM aims at improving access to quality health care. The programme integrates Family Welfare and National Disease Control Programmes, incorporates village health workers called Accredited Social Health Activists (ASHA) to the programme and forms Health and Sanitation Committees at village, block and district level.

The pilot phase of the NPCDCS was launched in Kerala (Thiruvananthapuram district) in 2008, with the aim to cover all districts by 2017. The NPCDCS combines population and high risk approaches. Mass media campaigns to raise public awareness are conducted by the state NCD cells. The high-risk approach includes opportunistic screening at health care settings, patient education and risk factor monitoring by nurses and health workers, lifestyle counseling by qualified counselors, and improved medical care and supervision by medical doctors [64]. Since the implementation is currently ongoing, no evaluations have been done so far. Legislation or recommendations and responsible implementation agents for tobacco use and diet and physical activity from the documents are listed in Table 2.

**Table 2:** Tobacco use, diet and physical activity recommendations and their implementation

**Year**	**Recommendations**	**Implementing agency**
**TOBACCO USE**		
2003	*Cigarettes and Other Tobacco Products Act (COTPA) 2003:*	The Department of Health and Family Welfare in each state is primarily responsible for implementation in coordination with other departments, authorised officers and various other stakeholders.
	- Prohibition of smoking in public places	
	- Prohibition of advertisement of cigarettes and other tobacco products	
	- Prohibition of sale of tobacco products to minors (below 18 years of age)	
	- Prohibition of sale of tobacco products by minors	
	- Prohibition of sale of tobacco products within 100 yards of the educational institutions	
	- Specified health warnings on tobacco products	
	- Testing of tobacco products for their harmful contents and emissions	
2007-2012	*Programme components of National Tobacco Control Programme (NTCP):*	NTCP to support implementation with national, state and district level actions and actors
	National level:	
	- Mass media campaigns to create public awareness	
	- Establishment of tobacco testing labs	
	- Mainstreaming the programme components as part of the health delivery mechanism under the overall NRHM framework	
	- Mainstreaming research and training on alternate crops and livelihoods and monitoring and evaluation including surveillance	
	State level:	
	- Establishment of a tobacco control cell	
	District level:	
	- Tobacco control centres	
	- Information, Education and Communication activities	
	- Training of professionals	
**DIET**		
2008 (pilot phase)	Guidelines by National Programme for Prevention and Control of Diabetes, Cardiovascular diseases and Stroke (NPDCS)*	Ministry of Health and Family Welfare, Govt. of India
	- Increase intake of green leafy vegetables and fresh fruits.	
	- Consume less salt; avoid adding or sprinkling salt to cooked and uncooked food.	
	- Preparations that are high in salt and need to be moderated are: Pickles, chutneys, sauces and ketchups, papads, chips and salted biscuits, cheese and salted butter, bakery products and dried salted fish.	
	- Restrict all forms of free sugars and refined carbohydrates for example biscuits, breads, naan, kulchas, cakes, and so on.	
	- Steamed and boiled food should be preferred over fried food.	
	- Have fresh lime water instead of carbonated drinks.	
	- Avoid eating fast or junk foods and aerated drinks. Instead of fried snacks, eat a fruit.	
	- In practice, it is best to use mixture of oils. Either buy different oils every month or cook different food items in different oils. Oils that can be mixed and matched are mustard oil, soya bean oil, groundnut oil, olive oil, sesame oil, and sunflower oil.	
	- Ghee, vanaspati, margarine, butter and coconut oil are harmful and should be moderated.	
	- If you are a non-vegetarian, try to take more of fish and chicken. They should not be fried. Red meat should be consumed in small quantities and less frequently.	
	- Eat variety of foods to ensure a balanced diet	
2010	*Guidelines by National Institute of Nutrition (NIN)*	These guidelines were proposed by the National Institute of Nutrition, Hyderabad which works under the aegis of Indian Council of Medical Research, Ministry of Health and Family Welfare, Govt. of India
	- Combine different food groups to obtain a well-balanced diet. Recommended balanced diet for adults with moderate physical activity (for reference men and women weighing 60 and 55 kg respectively): net energy (kcal/day): 2730 (men), 2230 (women); Fats and oils (visible fat): 5gX6 (men), 5gX5 (women); Sugar: 5gX6; Milk and milk products: 100gX3; Pulses: 30gX3 (men), 30gX2.5 (women); Vegetables (excluding roots and tubers): 100gX3; Fruits: 100gX1; Cereals and millets: 30gX15 (men), 30gX11 (women).	
	- Ensure provision of extra food and healthcare to pregnant and lactating women.	
	- Promote exclusive breastfeeding for six months and encourage breastfeeding till two years.	
	- Feed home based semi-solid foods to the infant after six months.	
	- Ensure adequate and appropriate diets for children and adolescents in health and sickness.	
	- Ensure moderate use of edible oils and animal foods and less use of ghee, vanaspati, and so on.	
	- Overeating should be avoided to prevent overweight and obesity.	
	- Restrict salt intake to minimum, should not exceed 6 g per day.	
	- Ensure safe and clean foods and practice right cooking methods and healthy eating habits.	
	- Drink plenty of water and take beverages in moderation. A normal healthy person needs to drink about 8 glasses (2 litre) of water per day.	
	- Minimize the use of processed foods rich in salt, sugar and fats. The intake of trans-fatty acids should not exceed 2% of energy intake.	
	- Include micronutrient rich foods in the diets of elderly people for them to be fit and active.	
	- Eat plenty of vegetables and fruits.	
	- Exercise regularly and be physically active to maintain ideal body weight.	
**PHYSICAL ACTIVITY**		
2008 (pilot phase)	Guidelines by the NPCDCS*	Ministry of Health and Family welfare, Govt. of India with WHO collaboration
	- Physical activity is a key determinant of energy expenditure.	
	- Regular exercise is important for promoting weight control or weight loss.	
	- Exercise regularly (moderate to vigorous) for 5–7 days per week; start slowly and work up gradually.	
	○ At least 30 min (accumulated) of physical activities per day for cardiovascular disease protection.	
	○ 45 min/day (accumulated) for fitness.	
	○ 60 min/day (accumulated) for weight reduction.	
	- Discourage spending long hours in front of television.	
	- Encourage outdoor activities like cycling, gardening and so on.	
	- A minimum 30–45 min brisk walk/physical activity of moderate intensity improves overall health.	
	- Include ‘warm-up’ and ‘cool- down’ periods, before and after exercise regimen.	
2010	*Guidelines by NIN*	Guidelines were proposed by the National Institute of Nutrition, Hyderabad which works under the aegis of Indian Council of Medical Research, Ministry of Health and Family Welfare, Govt. of India
		- Physical activity is essential to maintain ideal body weight by burning excess calories and is of vital significance for health and prevention of diseases.	
	- Physical activity is essential for the reduction of morbidity and mortality due to several chronic diseases and may reduce the risk of falls and injuries in the elderly.		
	- Exercise is a prescriptive medicine.		
	- Move your body as much as you can.		
	- Physical activity is a major modifiable risk factor in reduction of non-communicable chronic diseases.		
	- Recommended to carry out at least 45 min of moderate intensity activity, which may reduce the risk of chronic diseases.		
	- To lose weight 60 min of moderate to vigorous intensity physical activity may be taken for most of the days in a week.		
	- Children and teenagers need at least 60 min of physical activity every day. In the case of pregnant women 30 min or more of moderate-intensity physical activity every day is recommended.		

* The programme was launched in 10 states (including Kerala) and 10 districts as National Programme for Prevention and Control of Diabetes, Cardiovascular Disease and Stroke (NPDCS) in 2008. In 2010–11, when it was scaled up to 21 states and 21 districts, a cancer component was added and its name was changed to National Programme for Prevention and Control of Cardiovascular Disease, Diabetes, Cancer and Stroke (NPCDCS).

Together, these three – national policies addressing tobacco; the NRHM to improve access to health care; and the NPCDCS that includes broad approaches to prevention – provide a base of concerted government, health sector, and community campaigns to improve health care and to promote the personal behaviours linked to prevention and disease management.

### Focus groups

Content analysis revealed two main themes (Table 3): (1) What to intervene on with respect to behavioural targets and their determinants? (2) How to intervene in terms of preferences with respect to programme implementation? These themes and their sub-themes with views that emerged from the participants reveal a general interest to learn more and a common understanding that unhealthy lifestyle is related to a higher risk. However, they also showed misunderstandings concerning medication. Furthermore, perceptions related to risk as well as to outcomes of and self-efficacy in lifestyle change were generally low. Low male participation was not only an issue for the focus group study where most participants were females, but it was also indicated for village meetings, and the participants expected it to apply to any intervention as well.

**Table 3:** Responses of the focus group discussions as themes and sub-themes with descriptions

**Theme 1: What to intervene on with respect to behavioural targets and their determinants?**		**Theme 2: How to intervene in terms of preferences with respect to programme implementation?**	
**Sub-themes**	**Description and quotes**	**Sub-themes**	**Description and quotes**
1. Knowledge and beliefs of diabetes	General interest to know more about diabetes and its prevention.	1. Trusted sources of health information or potential intervention agents	· Health centers.
			· Physicians, health care providers.
			· Grass root level non-physician health workers.
			· Accredited Social Health Activists (ASHAs).
			· ‘*Kudumbasree*’ (local women’s self-help groups)
2. Risk factors	· Strong family history and the modern lifestyle.		
	· Unhealthy dietary habits including regular consumption of foods rich in fats and sugar like sweets, roots like tapioca and certain fruits, particularly sweet bananas like ‘*rasakathali*’. High consumption of pastries and snacks as parts of urban lifestyle.		
		2. Use and acceptance of Information Communication Technology (ICT)	· Telephone used by all and highly accepted for practical organization of meetings.
			· Mobile phones used and accepted for incoming calls.
	· Physical inactivity, particularly in sedentary occupations and in urban environment. “*People just don’t walk now-a-days*.”		· SMS used but not accepted.
			· Internet not used.
	· Long-term medications: “*Since I am on a lot of medication, I expect that I may get it. I have read in articles that those who take medicines for blood pressure and other things have a higher chance of getting such illnesses*.”		
	· Protective factors other than healthy food habits – e.g., “*using no sugar in tea for the last 15 years*” – rarely mentioned.		
3. Risk perception	· No awareness of pre-diabetes status.	3. Preferences for intervention delivery	*“If meeting points or places can be identified for each locality and the intervention is done as a group, it is better. It should be a place where people in that area can walk to or access easily.”* · Regarding low male participation to regular village meetings (*Gramsabha*), “*active men should invite other men*”. · Venue: a. Easy access. b. Within walking distance. c. Health centres, reading rooms or *anganwadis.* d. Participants’ homes. · Format: a. Group of 10–25 participants, important for generating different ideas that would benefit the whole group. b. Including at least two people per family and neighbours. Women might need permission from their husbands. · Time: a. Duration 1–1.5 h. b. Once-a-week. c. On holidays. d. When children are at school (for housewives).
	· Diabetes risk perceived higher for women, a group seen as less physically active, with a tendency to over eat and to ignore early symptoms.		
	· Perceived own risk: i. Little to no risk: Participants, who perceived their food habits were healthy; had no family history; or had faith that regardless of habits, they were simply not at risk. *“I don’t believe in any of this. I don’t feel I have any risk. I still need double sugar in my tea.”* ii. Fifty percent or more risk: Participants who already had a related illness like hypertension or myocardial infarction; or hypertensive or anti-cholesterol medication, perceived to contribute to high blood sugar; or who had significant family history. *“I expect a 50% risk as I am a hypertensive for the last 18 years and have been on medication and I had a heart attack 10 years back.”*iii. Don’t know: Not able or were not willing to speculate about their risk.		
4. Outcome expectations	· Diabetes has no cure, but can be controlled with oral medicines, injections, dietary and other lifestyle changes.		
	· Low outcome expectations for lifestyle modification after the pre-clinical or very early stages of the disease: *“You can only control it or decrease it. When food is controlled along with treatment, up to 80% can be controlled. Once you get the illness, you have no choice but to go for treatment.”*		
5. Self-efficacy	· A collective low self-efficacy regarding the ability to make and sustain changes in lifestyle. *“I don’t think it is possible to make modifications in our lifestyle. No matter what you say, it will just continue like this.”* · Dietary habits not within individual control. · Cultural norms such as “*fruits other than bananas belong to children’s diet only”* and collective household decision-making guide dietary practices. · Physical activity is related to everyday chores, like walking to the market, or to job like farm work, not to leisure-time. *“I am a driver working in the Gulf. When I come home for vacation, I do farming for four hours every day. I also have cows, so I get enough exercise. When I am in the Gulf, there is no time to walk or for any other exercise.”* For men, availability of time is a barrier while for women both time and space restrict the possibilities to be physically active. *“I used to do Yoga in the mornings. (…) When we go to the room, there should be no one else there. We need privacy. Slowly, it has become difficult to find such a time and space so, now I don’t do it anymore”. “I have heard that walking is good. But we have to start kitchen work at 6.30 in the morning, so when can we walk?” “We can’t go out of our own compound to walk. If we have space in our own backyard, it will work.”* · Quitting tobacco is hard because of social pressure. *“I have quit several times, each time for varying duration (…) Inevitably, I will see some of my friends smoking or they will offer a cigarette and I will start smoking again.”* · Professional help is not sought for quitting. *“If we want to stop smoking, we can decrease slowly not suddenly. If you smoke 10, you can make it 5, then 2 and then stop.”* · Women can only influence men’s use of tobacco by asking them not to smoke inside the homes.		

## Discussion

In this section, we provide a summary and synthesis of the findings, discuss the main strengths and limitations of the needs assessment, and outline the implications for designing a diabetes prevention programme for the rural setting of Kerala.

### Summary and synthesis of main findings

**Figure 1:**
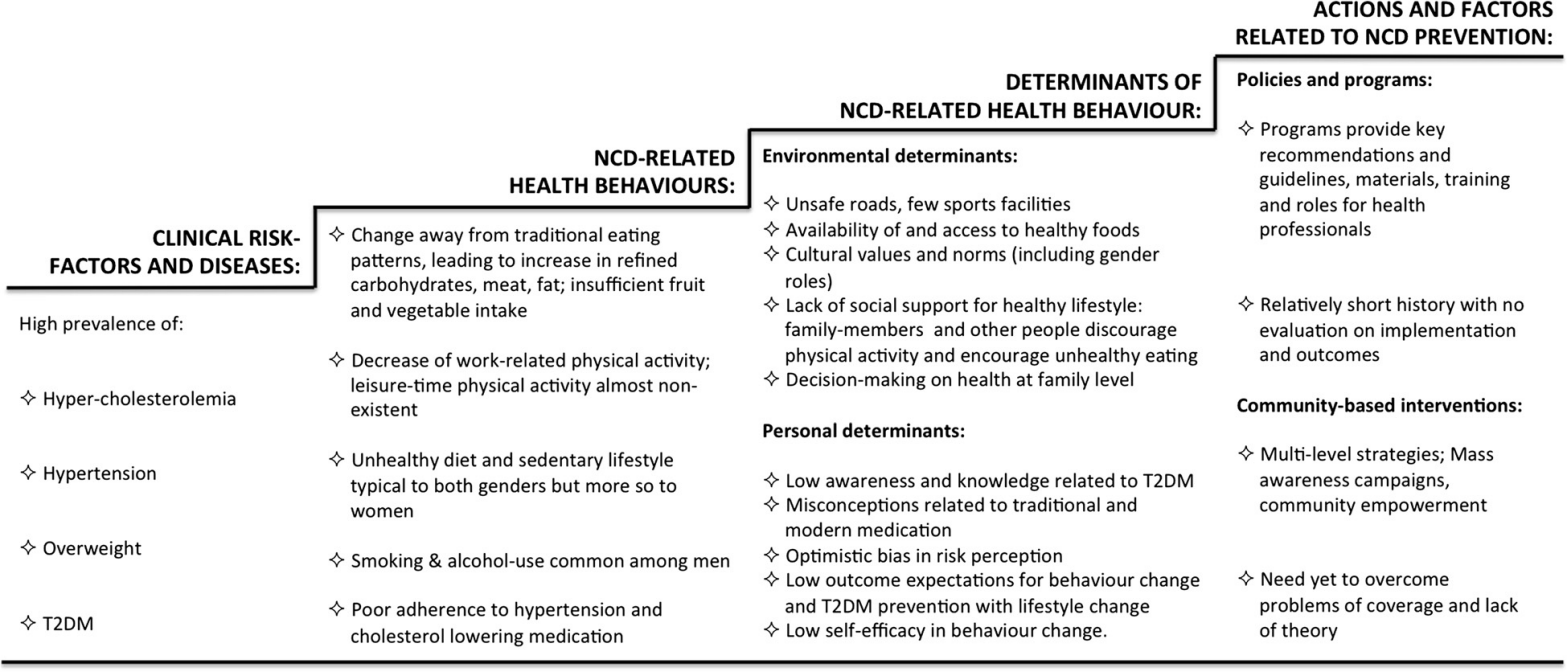
**Summary of findings from the needs assessment study.** This figure summarizes the findings of the needs assessment for the Kerala Diabetes Prevention Programme through triangulation and synthesis of evidence from three major sources of information: research literature review, policy document review and focus group study.

The findings on clinical risk factors and disease, and related lifestyle behaviours with their environmental and individual determinants, as well as main characteristics of current policies, programmes and interventions to prevent non-communicable diseases in India are summarised in Figure 1.

Prevalence of clinical and anthropometric risk factors is very high and increasing in the whole of India, and Kerala is the most advanced state in the transition. The changes are largely attributable to lifestyle, which is rapidly changing towards unhealthier patterns. While the unhealthy dietary patterns and sedentary lifestyle are typical for both genders, they are more common among women, which is reflected in women’s higher prevalence of T2DM [30]. Furthermore, sedentariness related to work, leisure-time and commuting applies not only in urban India but is also typical to the rural areas [39,65].

Many of the environmental determinants for unhealthy behaviours, e.g., accessibility, availability, and cultural values and norms are likely linked to each other. Furthermore, they probably contribute to many personal determinants such as low self-efficacy for behaviour change in the context of the strong influences of family decision-making and norms. Taking the environmental determinants as primary targets for intervention may not be feasible. However, the personal determinants are not sufficient targets alone, either. Rather, it may be unrealistic to expect better health behaviour outcomes, unless, the intervention addresses the influence of families and neighbours as well as the culturally inscribed roles of men and women in their families and communities.

Intervention research in India suggests that risk reduction is possible, but current evidence available from community settings is rather limited. In the absence of supports available for such programmes in developed countries (e.g., intensive schedules; special low calorie diets; extra resources for participants; free access to supporting facilities like gyms) [5,6], the risk reduction from intervention studies in India and similar settings will likely be more moderate. Previous research has established models in India for multi-level strategies including mass awareness campaigns, community empowerment, and individual empowerment [22,52-56,59]. However, the low reach of interventions in the community is a challenge, especially among those with higher needs, including rural people [55,66]. Another challenge is related to the use of relevant socio-behavioural theories, which is currently rare in interventions conducted in India. While studies from developed countries show that use of theory increases intervention effectiveness [57], the existing theories have been neither developed nor adequately tested with Indian populations to determine their usefulness in India. The qualitative findings from our needs assessment suggest that theoretical constructs such as self-efficacy and outcome expectations will likely have validity across cultures. However, their application in collectivist and family-oriented cultures will need further development in order for them to guide efforts at promoting behaviour change in India.

National programmes for tobacco control and prevention and control of major NCDs including T2DM have also been implemented in the state of Kerala. While the concerned government bodies have taken disease prevalence and morbidity and mortality patterns into consideration, the scientific basis for the proposed policies and programmes is not very clear in any of the accessed programme or policy documents. Moreover, their effectiveness has not been evaluated, thus limiting their usefulness to that of a positive reinforcer due to the launch of the mass awareness campaigns. In any case, these developments have prepared the ground for the development of NCD prevention programmes as well as promoting awareness among population and health professionals. In addition, their organizational infrastructure may provide local level resources to support intervention implementation by integration of many local agents such as village committees and village health workers like the ASHAs.

### Strengths and limitations

This study not only provides a quite comprehensive assessment of needs but also of available capacities and supports for T2DM prevention in Kerala. The findings are derived from a systematic review of major intervention studies as well as epidemiological research on social and behavioural risk factors conducted in India. The evidence is triangulated with two other major sources of information, that is, a review of policy development in relation to the prevention and control of NCDs as well as, a focus group study. However, the search for relevant policy documents was confined to the Internet and it focuses primarily on national policies and programmes. Where possible, local experts in Kerala were used to fill any gaps in relation to policies developed and implemented in Kerala. Although the small number of focus groups and low participation rate is an important limitation, there was good consensus between our major findings and those of other qualitative and quantitative studies having bigger samples, thereby increasing the external validity of our findings [46-49].

### Implications for developing the community-based K-DPP

#### Selection of behavioural targets

Increasing the consumption of fruits and vegetables and reducing sugar-containing foods are important targets for diet. The concept that physical activity is an integral and vital part of all aspects of daily life, with contexts other than just the work environment, also needs to be promoted. Earlier T2DM programmes from developed countries did not focus on tobacco use because that was not yet recognised as a risk factor to T2DM. However, with the present knowledge [67] and high prevalence of tobacco use among men in Kerala, it needs to be addressed in K-DPP. Furthermore, proper use of medication and traditional remedies are also important behavioural issues to address.

#### Main determinants to be addressed

Risk perception and knowledge about risk factors need to be core starting points for the intervention, as both low awareness and important misconceptions related to these factors have been identified. Based on our results, it seems clear that little standard information is currently made available to people in India concerning risk of diabetes, its causes and what to do about this in order to reduce risk of progression to diabetes and its complications. Focus group responses indicated that many people perceived that progression to diabetes and going on to medications was inevitable, due to family and cultural influences, and hence, it was not really possible for the individual to do anything about this. A diabetes prevention programme should educate those at risk and their families regarding these issues and the importance of a healthy diet and physical activity for both improved prevention and control of diabetes.

When considering the psychosocial determinants of lifestyle change, it is important to emphasise the ‘social’ aspect and to adopt a more family- and community-oriented approach rather than the more individualistic approach, which has been generally used in North American and European programmes. Given the very high prevalence of hypertension, hypercholesterolemia and other associated cardio-metabolic conditions, a key message should also be that improving diet, increasing physical activity and reducing sedentariness to prevent diabetes are good health habits for the entire family to reduce risk of chronic conditions, and that this approach is not just relevant to the individual at risk of diabetes. When assessing risks and reappraising outcomes, participants should be helped to consider the implications for their families, and to set goals together with their family and others living in their household. Instead of focusing on individual control, the issues of over-eating and physical inactivity, and the identification of healthier lifestyle options need to be addressed collectively. By emphasising collective problem solving, i.e., by finding solutions to key barriers together, and by identifying collective ways for pursuing a healthier lifestyle and for providing social support to one another, individuals’ low self-efficacy could be significantly enhanced. Community empowerment, which includes identification of key community leaders, citizens, organizations, volunteers and other resources, and supporting their role in creating social norms; and planning and developing local environments that would enable more healthy lifestyles will also, be an important enabler for individual empowerment.

### Intervention programme delivery

A group format for programme delivery was emphasised among our participants due to practical reasons but also as a supportive measure. Groups can integrate naturally occurring societal influences into the programme and provide channels for development of key processes within communities [68]. However, this kind of family- and community-oriented approach will also inevitably require making at least some of the group sessions available to other family members and neighbours to attend and to be involved. Additionally, peer-to-peer influences could be developed naturally to provide encouragement and assistance in reaching those who otherwise might not avail themselves of the programme [68], especially males. Low participation by males is a major threat to this approach and to address this, will require effective strategies for recruiting and maximizing the attractiveness of this kind of intervention approach for them. All of these factors provide a strong rationale for a peer support model as has been recently proposed [68].

#### Overall design of the study

The intervention for the Kerala Diabetes Prevention Programme is being designed with a theoretical base (theories of self-regulation and social support [16,21,68]), which has been shown to improve the effectiveness of interventions. Due to the relatively sparse evidence related to community-based NCD intervention research in India, many of the proposed strategies are being adapted from relevant intervention programmes undertaken in other countries that include China, United States, Australia and Finland. However, we are now able to combine these with the key elements and steps generated from our systematic review and analysis of the Indian and Kerala contexts in particular; and the various community meetings that have been held to discuss these issues during the recent piloting phase of K-DPP. However, the proposed approach will emphasize community empowerment as this has emerged both from the literature review and the focus group study as being very important. The strategies for the intervention programme are being currently piloted and further modified before the main trial commences. Finally, the rapidly evolving national and state policies and programmes focused on NCD prevention and control are also expected to provide support for K-DPP by raising awareness. Having a shared mission and understanding of the primary goals and strategies of any intervention programme with the local agents including health professionals, will also help to improve the programme reach among those with the highest need for the K-DPP intervention; as well as to ensure support for the implementation and long-term sustainability of the programme.

## Conclusions

The findings of this needs assessment study provide a strong basis for community-based diabetes prevention in the rural setting and culture of Kerala. They emphasize the significance of identifying key behavioural targets and their determinants; and to the importance of addressing cultural, community and family level factors in contrast to a more individualistic approach. They also suggest a more collectivistic approach for intervention delivery, with strong involvement of those at risk as well as their family and community members. These strategies along with the evolving support to be provided by national and regional policies and programmes to improve the prevention and control of NCDs will help the K-DPP achieve its goals as a feasible and effective intervention to prevent T2DM in rural India.

## Abbreviations

K-DPP: Kerala Diabetes Prevention Programme; T2DM: Type 2 Diabetes Mellitus; FGD: Focus group discussion; NCD: Non-communicable diseases; NRHM: National Rural Health Mission; NPCDCS: National Programme for control and prevention of Cancer, Diabetes, Cardiovascular Diseases and Stroke; ASHA: Accredited Social Health Activist.

## Competing interests

The authors declare that they have no competing interests.

## Authors’ contribution

MD: contributed to the conception and design of the study, conducted the research literature and policy document review, FGD data collection and analysis, conducted the evidence triangulation and synthesis, drafted as well as revised the manuscript; PA: conceptualised and designed the study, conducted the research literature and policy document review, conducted the evidence triangulation and synthesis, drafted as well as revised the manuscript; ST: conducted the research literature review, participated in evidence triangulation and synthesis, and revision of the manuscript; KRT: contributed to the conception and design of the study, participated in evidence triangulation and synthesis, and critically revised the manuscript; EBF: contributed to the conception and design of the study, participated in evidence triangulation and synthesis and critically revised the manuscript; NEP: participated in evidence triangulation and synthesis and revision of the manuscript; EM: participated in evidence triangulation and synthesis and revision of the manuscript; BO: conceptualised and designed the study, participated in evidence triangulation and synthesis, critically revised the manuscript and gave final approval of the version to be published. All authors read and approved the final manuscript.

## Pre-publication history

The pre-publication history for this paper can be accessed here:

http://www.biomedcentral.com/1471-2458/13/95/prepub

## Supplementary Material

Additional file 1 Criteria and search strategy used for research literature search. Click here for file

Additional file 2 Kerala Diabetes Prevention Programme - focus group questions. Click here for file
