# Supplementary material for: Lifestyle change in Kerala, India: needs assessment and planning for a community-based diabetes prevention trial
Source: BMC Public Health. 2013 Feb 1;13:95. doi: 10.1186/1471-2458-13-95 (PMC3576354; doi:10.1186/1471-2458-13-95)
Supplement: Additional file 1 — Criteria and search strategy used for research literature search. [file 1471-2458-13-95-S1.pdf]

Appendix 1: Criteria and search strategy used for research literature search.

Four searches were conducted in PubMed database using the following search strategies:

1. (("Diet"[Mesh] OR "Diet Therapy"[Mesh] OR "Food Habits"[Mesh]) OR ("Nutrition Assessment"[Mesh] OR "Nutrition Therapy"[Mesh] OR "Nutrition Policy"[Mesh])) AND "India"[Mesh] AND (English[lang] AND ("young adult"[MeSH Terms] OR "adult"[MeSH Terms:noexp] OR "middle aged"[MeSH Terms] OR ("middle aged"[MeSH Terms] OR "aged"[MeSH Terms]) OR "aged"[MeSH Terms] OR "aged, 80 and over"[MeSH Terms]) AND "2002/04/06"[PDat] : "2012/04/02"[PDat])

Total articles: 302

2. (("Life Style"[Mesh] OR "Risk Reduction Behavior"[Mesh]) AND ("Diabetes Mellitus"[Mesh] OR "Diabetes Mellitus, Type 2"[Mesh] OR "Cardiovascular Diseases"[Mesh])) AND "India"[Mesh] AND (English[lang] AND ("young adult"[MeSH Terms] OR "adult"[MeSH Terms:noexp] OR "middle aged"[MeSH Terms] OR ("middle aged"[MeSH Terms] OR "aged"[MeSH Terms]) OR "aged"[MeSH Terms] OR "aged, 80 and over"[MeSH Terms]) AND "2002/04/06"[PDat] : "2012/04/02"[PDat])

Total articles: 62

3) "Health Promotion"[Mesh] AND "India"[Mesh] AND (English[lang] AND ("young adult"[MeSH Terms] OR "adult"[MeSH Terms:noexp] OR "middle aged"[MeSH Terms] OR ("middle aged"[MeSH Terms] OR "aged"[MeSH Terms]) OR "aged"[MeSH Terms] OR "aged, 80 and over"[MeSH Terms]) AND "2002/04/06"[PDat] : "2012/04/02"[PDat])

Total articles: 85

4. ("Tobacco Use Cessation"[Mesh] OR "Tobacco, Smokeless"[Mesh] OR "Smoking"[Mesh]) AND "India"[Mesh] AND (English[lang] AND ("young adult"[MeSH Terms] OR "adult"[MeSH Terms:noexp] OR "middle aged"[MeSH Terms] OR ("middle aged"[MeSH Terms] OR "aged"[MeSH Terms]) OR "aged"[MeSH Terms] OR "aged, 80 and over"[MeSH Terms]) AND "2002/04/06"[PDat] : "2012/04/02"[PDat])

Total articles: 332

All available abstracts were reviewed and relevant articles were obtained through internet or directly from the authors. For the systematic review on interventions related to NCDs in the Indian context, we applied the following inclusion criteria: Dietary, Physical activity, tobacco-related or lifestyle modification interventions conducted in India among adult population (>/= 19 years) looking at individual risk factor modification alone or in combination with life-style modification for diabetes, cardiovascular or non-communicable diseases and published in the last 10 years. Eleven studies were identified that fulfilled the inclusion criteria, of which nine could be accessed; and these are described in Table 1.
