# Supplementary material for: Lifestyle change in Kerala, India: needs assessment and planning for a community-based diabetes prevention trial
Source: BMC Public Health. 2013 Feb 1;13:95. doi: 10.1186/1471-2458-13-95 (PMC3576354; doi:10.1186/1471-2458-13-95)
Supplement: Additional file 2 — Kerala Diabetes Prevention Programme - focus group questions. [file 1471-2458-13-95-S2.pdf]

APPENDIX 2
KERALA DIABETES PREVENTION PROGRAM
FOCUS GROUP QUESTIONS

The questions for the FGD were grouped into four sections as given below. The sections I to III deal with questions that were asked during the first two focus group discussions, while section IV describes the questions that were addressed in the third FGD.

Focus Group Discussions 1 & 2

I. Understanding of diabetes

These questions are to help us better understand peoples' knowledge, attitudes or beliefs and behaviours in relation to diabetes, its prevention and management/treatment

1. What do you think about diabetes? What kind of a disease is diabetes in your opinion? Do you think diabetes is a serious disease?
2. What do you think about the curability of diabetes? Do you think diabetes can be cured?
3. What do you think are the causes of diabetes?
4. What do you think are the behaviours or risk factors that lead to risk of diabetes?
5. What kind of people do you think are at risk for diabetes? What makes them be at risk? What do you think about your own risk? Do you think you are at risk for diabetes? What kinds of treatments do you think there are for diabetes?
6. Has anyone ever told you that you are at high risk of developing diabetes?
7. Is there something you would like to learn about diabetes? What? Would you like to be more educated about diabetes?
8. What would you like to know about diabetes prevention?
9. Have you or anyone in your family previously had diabetes?

II. Health information and access

These questions are about how people access information and which sources they trust.

10. If you or one of your friends wanted to lose weight, or become more physically active to prevent diabetes, how would they do it? What help could they get?
11. If you were looking for information about your health, where would you look for information? (e.g. internet, doctor, friends, family)
12. Are there any barriers for you in seeking healthcare? What?

III. The intervention or program and its delivery

These questions are to 'test' interest in, feasibility and acceptability of the kind of intervention we've proposed with the content and means of delivery described in our application.

13. If you were found at risk for diabetes, would you be willing to learn how to prevent it?
14. Would you be willing to participate in a program to help you eat more healthy foods, exercise more and (possibly) prevent diabetes?
15. If you know you could improve your lifestyle to be healthier and prevent diabetes, what do you think would get in the way of making healthy changes?
    a. What would get in the way of eating a more healthy diet
    b. What would get in the way of quitting smoking
    c. What would get in that way of doing more exercise

(E.g. might be family, work, difficulty in finding healthy food, time limitations)

16. If you were to participate in a program to assist you in eating a healthy diet, exercising more and losing weight, how would you most like the program to run? For example, would you prefer to:
    a. See a health professional on a regular basis (would you like to do this in your home or in the community? Which kind of professionals e.g. doctor, nurse, other?
    b. Take a partner or family member to see a health professional
    c. Have a health professional visit your whole family
    d. Meeting in a community group: if so, would you like to take a family member along?
    e. Receive information over the phone
    f. Receive text messages or emails
    g. Have access to advice on an internet based-system
17. If you were to participate in a program to assist you in preventing diabetes, how much time would you be prepared to do this....and what would be the most appropriate time to attend sessions? i.e. Weekday, night time, weekends etc?
18. If you were to participate in a program to assist you in making health changes, who would you like to receive the information from? (examples might be doctor, teacher, nurse, trained village health worker, other)
19. If you were to participate in a program to help you that was run by a community health worker and several lay people training to help their neighbourhoods, what would you think would be important to make the program helpful?
20. If you were to participate in a program to help you eat a healthier diet and be more physically active and lose weight and the program were run by a community health worker and several lay people trained to help their neighbours in this way, what would you think about various ways the program might be organized – primarily in group meetings with other friends or neighbours or primarily through individual education,
21. It’s hard to stay motivated to eat healthy and be physically active. What could a program like we’ve discussed do to help you in this? What could the community health worker and lay people working with the program do to help you stay motivated?
22. Do you have access to the internet?
23. Do you have access to a mobile phone? Do you feel comfortable using SMS?
24. Do you have a landline phone?
25. Do you have access to transport to attend a program that is not in walking distance?
26. As part of this program, would you be willing to have clinical measures taken (e.g. blood sample) for us to see your improvements?

Focus Group 3

IV. The 'How' of intervention delivery

The major purpose of this 3rd group with 'high risk' or pre-diabetes individuals is to build on the findings from the first two focus groups and to understand more about "how" the program could be delivered in ways that will be acceptable, feasible and culturally appropriate to the Kerala population.

27. How should each of the intervention groups be organized? What kind of venue would you prefer? What do you feel is the best way to carry out these sessions?

28. How should the "curriculum" be delivered? (Should it be structured or interactive or somewhere in between?).

29. The total period of the planned intervention is 3 months in the first instance but many groups will continue after that and might develop in other ways. What periodicity of sessions would you prefer? (? Once a week,? 12 sessions, etc) What should be the duration and timing of each session?

30. What can we do to engage the men? How can they be encouraged to participate actively and to attend all the sessions?

31. From current literature, we know that to reduce the risk of diabetes, we need to increase fruit and vegetable intake, increase physical activity, decrease tobacco use, decrease waist circumference (alcohol issue will definitely come up, so, it will have to be addressed as well). We also know that if lifestyle is modified; there is also economic relief, owing to better health, postponing the advent of NCDs, etc. How can we help you achieve this? With your dietary patterns and cultural background, how can we do this? What is going to be most practically helpful for you? What would you particularly like to know or learn?

32. How should the issue of nutrition be addressed? Cooking classes? Demonstrations? Session with a dietician?

33. Physical activity: What kind of physical activity would be preferred? What would be acceptable to your spouse or families? What would be practically possible? What about joint activity with your spouse? Group activity? What about the use of pedometers? Would you be interested?

34. How about tobacco and alcohol use? How can such a program help you as well…

35. Making a few small changes in your health habits is always worthwhile – every little bit helps. However, if you are at high risk for diabetes and want to prevent becoming diabetic, you really need to make some substantial changes. You will need to lose some weight (probably about 5 to 10% of your weight) and increase your physical activity to about 150 minutes a week – that's 5 days, 30 minutes a day. To help you accomplish these, a program would have to push you to set some goals for changing your eating and exercise habits and for losing weight and then would have to follow up with you to encourage you to do these things.
    How do you feel about this? How would a program best do this?
    Would you feel comfortable receiving this kind of encouragement to change your eating and activity patterns in a group setting?
